# First Report of Microplastics in Wild Long-Tailed Macaque (*Macaca fascicularis*) Feces at Kosumpee Forest Park, Maha Sarakham, Thailand

**DOI:** 10.3390/vetsci11120642

**Published:** 2024-12-11

**Authors:** Penkhae Thamsenanupap, Natapol Pumipuntu, Tawatchai Tanee, Pensri Kyes, Apichat Karaket, Randall C. Kyes

**Affiliations:** 1 Faculty of Environment and Resource Studies, Mahasarakham University, Maha Sarakham 44150, Thailand; penkhae.t@msu.ac.th (P.T.); tawatchai.t@msu.ac.th (T.T.); 2 One Health Research Unit, Mahasarakham University, Maha Sarakham 44000, Thailand; 3 Faculty of Veterinary Sciences, Mahasarakham University, Maha Sarakham 44000, Thailand; 4 Department of Psychology, Center for Global Field Study, and Washington National Primate Research Center, University of Washington, Seattle, WA 98195, USA; pkyes@uw.edu; 5 Department of National Parks, Wildlife and Plant Conservation, Bangkok 10900, Thailand; yo.forest@hotmail.com; 6 Departments of Psychology, Global Health, and Anthropology, Center for Global Field Study, and Washington National Primate Research Center, University of Washington, Seattle, WA 98195, USA; rkyes@uw.edu

**Keywords:** Kosumpee Forest Park, long-tailed macaques, microplastics, pollution, wildlife

## Abstract

Microplastic pollution is a global concern arising from the extensive production and use of plastics. The prevalence of microplastics (MPs) in the environment is escalating due in large part to the excessive use of plastics in various human-related activities. Consequently, animals are being exposed to MPs through dietary intake, which poses significant health risks to the wild populations. The objective of the study was to assess the concentration of MPs in the feces of wild long-tailed macaques (*Macaca fascicularis*) in the Kosumpee Forest Park (KFP) located in Northeast Thailand. KFP is situated in close proximity to the town of Kosum Phisai and experiences considerable human–primate interaction. Fresh fecal drops from 50 adult macaques were collected and sampled. MP presence in the feces was measured using density separation through visual identification under a stereomicroscope. We found a total of 396 MP particles in the feces with an average of 7.9 particles/macaque. Two forms of MPs were found in the macaques’ feces including fibers (391 pieces; 98.73%) and asymmetric fragments (5 pieces; 1.27%), with sizes mostly ranging under 1000 µm. The most observed color of MPs was blue (152 pieces; 38.48%). This study highlights the impact of anthropogenic waste and the potential health problems that can be caused to wild animals via microplastic pollution. The results contribute to the ongoing discussions on environmental health within the One Health framework.

## Simple Summary

Plastic pollution is a growing environmental concern, and microplastics are increasingly being detected in wildlife. This study focused on long-tailed macaques living in the Kosumpee Forest Park, Thailand, which are frequently exposed to human activities. We investigated the presence of microplastics in the macaques’ feces to understand the extent of contamination and to assess the macaques’ potential as bioindicators for environmental pollution. Our findings revealed microplastic contamination in all the sampled macaques. Fibrous particles were the most common type, and blue particles were the most prevalent color. These results highlight the significant impact of human activities, such as agricultural practices and waste management, within the forest park ecosystem. By studying microplastic levels in wildlife, we gain valuable insights into how these pollutants move through the ecosystems shared by animals and humans. This research emphasizes the need for better strategies to reduce plastic pollution and protect both wildlife and human health, contributing to a broader understanding of the environmental risks and solutions.

## 1. Introduction

Over the past decade, the pervasive issue of microplastic contamination has emerged as a critical concern in environmental, animal, and public health [1]. Microplastics (MPs), defined as plastic particles that are equal or less than 5 mm in size, have infiltrated ecosystems worldwide, posing a substantial threat and significantly impacting various animal species across different ecosystems, both terrestrial and aquatic [2]. MPs originate from a variety of sources, such as the degradation of larger plastic items, including bottles and packaging, as well as from the release of micro-sized plastic beads found in personal care products. These sources are primarily classified into the terrestrial and marine environments [3,4,5]. The use of granules and resin pellets in industrial activities also contributes to MP pollution in water bodies [6]. Additionally, personal care products, including soaps, toothpaste, shower gels, face wash foam, and laundry detergents, also contain MPs as drug carriers or ingredients [7]. Therefore, it is essential to study the distribution of MPs in the environment and find effective control measures to help mitigate these various sources of pollution.

MP particles infiltrating soil and water pose serious threats to many living organisms [8]. MPs can degrade into nanoplastics and even smaller particles that are comparable in size to bacteria and viruses, thereby increasing health risks [1,3,9]. Both direct ingestion and consumption through the food chain as bioaccumulation and biomagnification can result in MP accumulation in the tissues, particularly in the digestive systems of animals and humans [5,6]. These particles are often excreted, but some of these tiny particles can enter the bloodstream, obstructing blood flow and potentially leading to cancer by binding to blood cells and compromising the immune system [6]. Addressing plastic pollution is therefore crucial to protecting both animal and human health [9].

Kosumpee Forest Park, located in Kosum Phisai District, Maha Sarakham province, is a significant tourist attraction in the northeastern region of Thailand and home to a population of wild long-tailed macaques (*Macaca fascicularis*). This long-tailed macaque population has experienced considerable growth over the past 35 years [10], with a population estimated at 940 macaques at the time of this study (Tanee, personal communication). The macaques’ habits, including their daily activity patterns such as foraging behavior, are closely influenced by both the natural and anthropogenic environments. Their diet includes natural food sources within the forest park (e.g., fruit, leaves, insects), agricultural crops adjacent to the park (e.g., rice), as well as substantial provisioning provided by the park staff, locals, and tourists. These provisioned items include a wide variety of vegetables, fruits, snacks (e.g., chips, cookies, breads, etc.), food scraps, fruit juice, soft drinks, etc. Frequently, the natural and provisioned food sources are insufficient to meet the daily dietary needs of this macaque population, prompting them to forage through refuse for additional food scraps [11]. This foraging behavior significantly increases their exposure to microplastics both directly and indirectly, as has been noted in the previous research [12]. The macaques have access to a variety of water sources in and around the park, including the Chi River that borders the park, small water bodies within the park, and wastewater treatment ponds located adjacent to the park that process effluent from human activities, including those from the surrounding community and Kosum Phisai Hospital.

Given the increasing amount of plastics present in the environment, it is clear that understanding the impact of microplastics on animal health (including wildlife) is essential for a comprehensive One Health approach [6]. Recognizing the urgency of this issue, the objective of our study was to investigate and quantify the accumulation of microplastics in macaque feces within the Kosumpee Forest Park vicinity. We hypothesize that long-tailed macaques (*Macaca fascicularis*) can serve as a sentinel species for MP pollution due to their behavioral ecology, which is influenced by both natural and anthropogenic environments. We expect to find evidence of MP accumulation in the feces, reflecting the environmental contamination of their habitat, that is, the result of human activities. These data will not only provide a baseline measure for assessing environmental MP levels but also will serve as a guide in developing effective strategies for managing and mitigating microplastic contamination in the environment.

## 2. Materials and Methods

### 2.1. Study Sites

**Figure 1:**
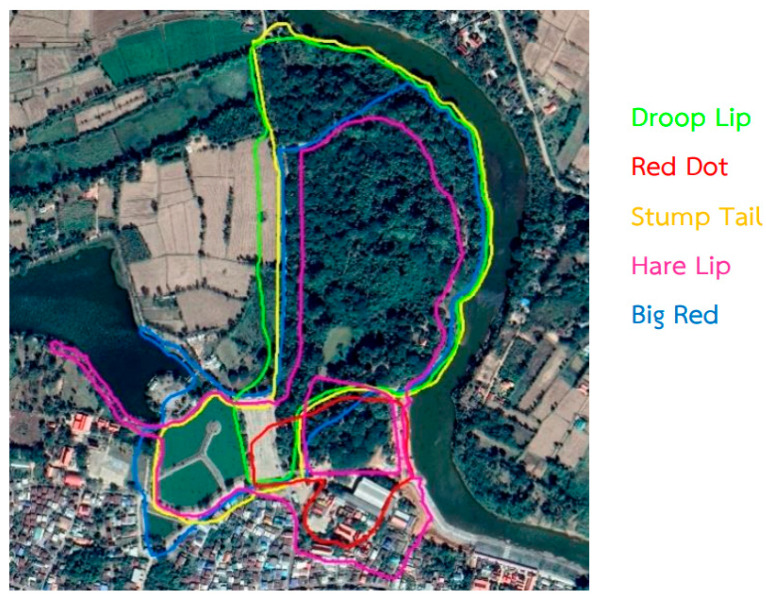
Kosumpee Forest Park study site and the estimated home range of each group of macaques. (Adapted from Kyes, et al., 2018 [10]).

The study was conducted at the Kosumpee Forest Park (N16°15′12.6″ E103°04′02.0″). Kosumpee Forest Park (KFP) is located in Maha Sarakham Province, Northeastern Thailand (Figure 1). The forest park consists of a fragmented forest patch of approximately 0.2 km^2^, bordered on the east by the Chi River, by agricultural fields to the west and north, and by the town of Kosum Phisai to the south [13]. The long-tailed macaque population in KFP has grown considerably over the years. By the end of the year 2016, the macaque population was reported to be more than 730 macaques distributed among five social groups, with largely overlapping home ranges [10]. That number increased to over 940 macaques by the time of this study in 2019 (Tanee, personal communication). In KFP, as in many other locations, the locals and macaques have a high level of interaction, due to urban and agricultural expansion. As such, the macaques are exposed to a range of anthropogenic pressures and waste [14]. The macaques in the KFP drink water from 3 major sources, including the Chi River, a pond in the forest park, and a municipal wastewater treatment pond close to the park. These three sources of water are situated close to the public and are polluted with plastic waste.

### 2.2. Sample Collection

Fecal samples were collected from 50 adult macaques in January 2019. Ten macaques were sampled from each of the five groups (including DroopLip, RedDot, StumpTail, HareLip, and BigRed as shown in Figure 1) to obtain a representative sample of the population. These macaques were identified using facial features and unique characteristics, following a photographic directory created by Kyes et al. [10]. Fresh fecal drops from identified macaques were immediately collected from the ground, place into collection glass tubes containing formalin, sealed, labeled and transported to the laboratory for processing. All the samples were preserved at 4 °C for subsequent analysis.

### 2.3. Sample Processing

Fecal sample processing followed the protocol by Thamsenanupap et al. [15]. The samples were digested in 30% hydrogen peroxide (H_2_O_2_) solution for 12 h and then filtrated using a 0.47 µm pore size Whatman GFC filter. The samples were then placed on covered glass plates and dried in an oven at 65 °C for 4 h prior to MP analysis.

A total of 10 g of each fecal sample was mixed with 200 mL of saturated sodium chloride solution, stirred at 200 rpm for 2 min, and then left to settle. The water layer was sieved through the 850 and 600 µm pore size Whatman GFC filters and then filtered using a 0.47 µm pore size Whatman GFC filter. The remaining sample was mixed with 375 mL of 60% sodium iodine solution, stirred at 200 rpm for 2 min, and then left to settle for 10 min. Water with the remaining MP particles was filtered using a 0.47 µm pore size Whatman GFC filter. The accumulated MPs on the filter were treated with 30% H_2_O_2_ solution.

### 2.4. Analysis

Microplastic observation and analysis was conducted under a stereomicroscope (Zeiss Stemi 305 Standard K-Lab, Oberkochen, Deutschland). Under the stereomicroscope, all MP samples were examined and photographed using a digital camera (Cannon EOS 800D, Tokyo, Japan) with AxioVision LE image analysis software (version 4.8, Carl Zeiss Microscopy, White Plains, NY, USA). MP abundance, color, and morphology (fiber, fragment, pellet or foam, non-patterned or irregularly shaped fragments) were identified visually if they satisfied the characteristics described in Thamsenanupap et al. [15]. To control for MP contamination during sample collection, processing, and analysis, a combination of measures was implemented including the use of glassware instead of plastic, regular cleaning of the lab area, working in a fume hood, and wearing cotton clothing. All sample analysis was conducted using laminar flow cabinets equipped with HEPA filters to minimize the risk of contamination.

### 2.5. Data Analysis

Descriptive statistics were used to describe the occurrence and quantity of MPs in the long-tailed macaques at the KFP. A confidence interval of 95% and the proportion of MPs quantification were used to assess the data. A chi-squared test and Fisher’s exact test of independence were performed (using SPSS, version 22) to analyse potential differences between the five groups of macaques. Statistical significance was determined using a probability value (*p*-value) < 0.05.

## 3. Results and Discussion

### 3.1. Abundance of Microplastics in Macaques’ Feces

**Figure 2:**
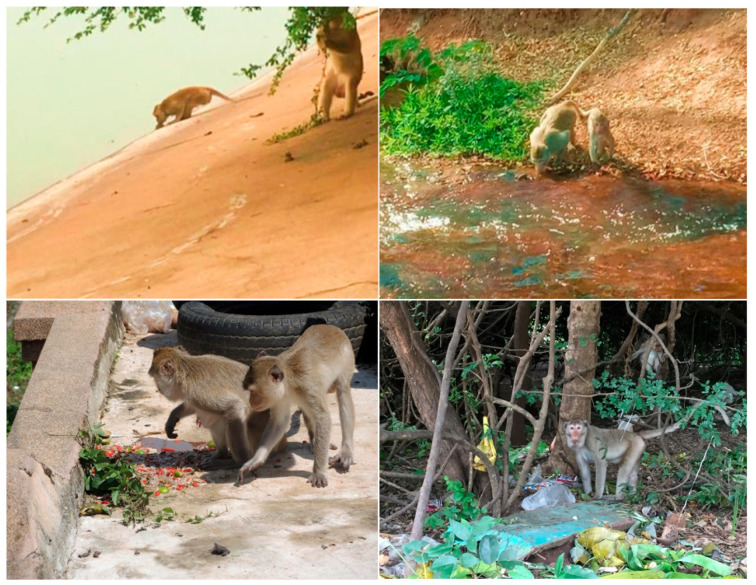
Images of macaques drinking from water sources and foraging on garbage from the community—potential sources for ingesting MPs.

Based on analysis of the fecal samples collected from the 50 macaques at the Kosumpee Forest Park, a total of 396 potential MP particles were discovered. Microplastics were found in every macaque, with an average of 7.9 particles per macaque (range: 1 to 27 particles per individual). Given that the five groups of macaques inhabited a small forest area with largely overlapping home ranges with similar exposure to sources of MPs (Figure 2), potential differences in MP contamination among the groups were not expected. We assessed that the MP concentration was highest in the StumpTail group, with an average of 12.4 particles per animal, whereas the HareLip group exhibited the lowest concentration, averaging 3.1 particles per animal, as shown in Table 1. We assume this difference is attributable to the limited sample size per group. As such, we believe the results are best interpreted at the population level.

Our current study provides significant insight into the accumulation of microplastics in wild long-tailed macaques both within and beyond the boundaries of KFP. To the best of our knowledge, this study is the first to demonstrate (via fecal analysis) the ingestion of MPs by non-human primates in Thailand. These findings underscore the significance of MP contamination in terrestrial ecosystems, an aspect that has been comparatively underexplored. Previous research has predominantly focused on freshwater fish, edible arthropods, and aquatic environments in Northeastern Thailand [15,16,17].

These findings add to the growing body of literature that indicates that MPs are pervasive and widespread in terrestrial ecosystems. In the case of KFP, the unique geographical characteristics of the region suggest that MPs may be transported into the park via multiple pathways, including human water resources, the Chi River, wind, rainfall, garbage pollution, and food packaging. This highlights a potential “microplastics—human—animal” transport model within the ecosystem.

### 3.2. Morphology, Size, and Color of MPs in the Macaques’ Feces

**Figure 3:**
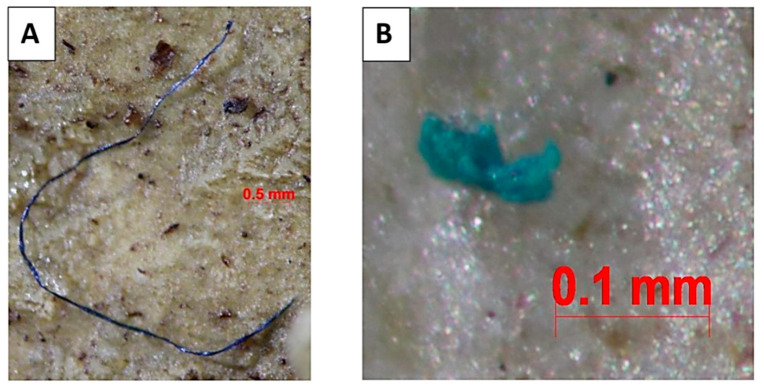
Morphology of microplastics in the macaques’ feces from Kosumpee Forest Park. (**A**) Fibrous microplastic, (**B**) non-patterned microplastic.

Analysis of the MP particles indicated the presence of the following two types: fibrous and non-patterned (Figure 3). Fibrous MPs were present in all macaques, whereas non-patterned microplastics were found only in some of the macaques. The highest concentrations of fibrous MPs were observed in the RedDot and BigRed groups, with both exhibiting 100% presence. This was followed by the DroopLip, StumpTail, and HareLip groups, respectively (Table 1).

Our study showed that the MPs identified were distributed across six size ranges, as shown in Table 2. A majority of the MPs (45.06%) were in the 0–1 mm range, with 178 pieces found. The 1–2 mm size accounted for 28.86% (114 pieces), followed by 13.17% (52 pieces) in the 2–3 mm range. The 3–4 mm and 4–5 mm sizes contributed 6.08% (24 pieces) and 4.81% (19 pieces), respectively, while the >5 mm category accounted for only 2.02% (8 pieces) of the MPs. These data indicate that smaller microplastics are more prevalent, likely due to their lighter weight, which facilitates their entry into the food chain.

**Figure 4:**
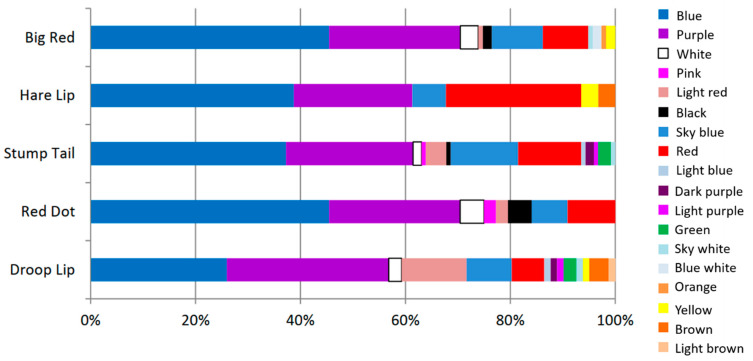
Color range of microplastics in the macaques’ feces from Kosumpee Forest Park. Note: Colors are intended to reflect the diversity of MPs that may originate from various plastic products and industries including fishing nets, ropes, or synthetic textiles, packaging, automotive parts, or industrial materials, plastic bottles or food packaging.

The MPs found in the macaques’ feces consisted of 18 distinct colors, as shown in Figure 4. This is likely to reflect a range of contamination sources, with the wastewater treatment ponds located near the forest park being a primary contributor. These ponds receive effluent from surrounding communities, where household activities such as laundry contribute microplastic fibers, particularly polyester, into the water system [18,19,20,21,22,23]. The most frequently detected microplastic color was blue, comprising 38.48% of the total MPs and present across all the macaque groups. Purple followed at 25.82%, while light brown was the least common at 0.25%.

The predominance of blue microplastics in the samples may be attributed to multiple factors. For example, blue is a common color in consumer goods, such as packaging and textiles, reflecting its aesthetic appeal and widespread production [21,24]. This aligns with patterns of human plastic usage. Additionally, blue microplastics, often derived from durable synthetic fibers, exhibit high environmental persistence due to their resistance to degradation [24].

It is well established that the presence of MPs in the environment poses significant health risks for both humans and wildlife, including macaques. In humans, MPs have been linked to oxidative stress, inflammation, and cytotoxicity, which can disrupt immune function, impair metabolism, and increase the risk of chronic diseases such as cancer and autoimmune disorders. MPs can translocate through the circulatory system, causing tissue accumulation, blockages, and potential developmental impacts, such as crossing the placental barrier. Similarly, in wildlife, MPs accumulate in the gastrointestinal tract, causing physical damage, reduced nutrient absorption, and behavioral disruptions. They also act as vectors for toxic chemicals like persistent organic pollutants (POPs) and heavy metals, leading to bioaccumulation and long-term health effects, including hormonal disruption and immune system impairment [25]. Macaques, as potential sentinel species, provide critical insights into the ecological and health implications of MP contamination, underscoring the need for further research on its cascading impacts within the ecosystems shared by humans and wildlife.

Our research has shown that the prevalence of MPs in the feces of the long-tailed macaques at KFP is high. The findings provide preliminary insight into the presence of MPs in wild terrestrial vertebrates and build on the other recent studies in Thailand and Southeast Asia [26]. Further, monitoring plastic pollution through fecal analysis offers a non-invasive and ethical approach for assessing MP excretion in wild animals [27,28,29,30,31,32,33,34]. The long-term effects of MPs on the long-tailed macaques in Kosumpee Forest park are of critical importance to our understanding of the potential, long-term health risks posed by the MPs and thus deserved further investigation.

When comparing our findings with a recent study of Yunnan snub-nosed monkeys (*Rhinopithecus bieti*) from the Southern Hengduan Mountains in China [35], we find valuable context for understanding the breadth of MP contamination in terrestrial wildlife. The Yunnan snub-nosed monkeys showed significantly higher MP content in their feces (75.263 ± 58.141 MPs/g) compared to our study, with an average of 7.9 MPs per individual. This contrast likely reflects the role of regional and anthropogenic factors, such as the differences in habitat characteristics, dietary sources, and human activities, in influencing MP exposure.

A limitation of our study is the focus on fecal samples as indicators of microplastic contamination, which may not fully represent the broader environmental or health impacts. Incorporating direct environmental sampling in future research could provide a more comprehensive view of contamination sources. Additionally, the lack of GPS tracking to accurately monitor macaque movements and overlaps limits the precision of group differentiation based on home range and may affect conclusions about contamination dynamics. Addressing these limitations in future studies will enhance the accuracy and scope of the findings.

## 4. Conclusions

This study highlights the prevalence of microplastics (MPs) in a terrestrial wildlife species, the long-tailed macaques, in the Kosumpee Forest Park, Maha Sarakham, Thailand. Based on an analysis of the macaques’ fecal samples, which showed a dominance of fibrous MPs and blue as the most common colour, our findings underscore the significant impact of anthropogenic activities on microplastic contamination in terrestrial ecosystems. To our knowledge, this is the first report on MP quantification in wild monkeys in Thailand. Further, this research represents one of only a handful of studies addressing MP contamination in wild primates in Southeast Asia and thus contributing to a growing body of knowledge on environmental pollution.

The use of fecal samples to detect MPs offers a non-invasive method to monitor contamination levels and trends over time, providing valuable data for understanding temporal variations in MP distribution within terrestrial ecosystems. Such insights can inform strategies to mitigate MP contamination and guide the development of regulations and policies aimed at protecting ecosystems and their vertebrate inhabitants. Further research is needed to evaluate the long-term ecological and health impacts of MPs on wildlife and the animals’ potential role as indicators of environmental contamination. Ultimately, these studies will contribute to a broader One Health approach.

## Figures and Tables

**Table 1:** Abundance and morphology of microplastics in the long-tailed macaques’ feces from Kosumpee Forest Park.

Macaque Group	Number of MPs (%)	Morphology of MPs (%)	
		Fibrous MPs	Non-Patterned MPs
DroopLip	81 (20.46)	79 (97.53)	2 (2.47)
RedDot	44 (11.11)	44 (100)	-
StumpTail	124 (31.31)	123 (99.19)	1 (0.81)
HareLip	31 (7.83)	29 (93.55)	2 (6.45)
BigRed	116 (29.29)	116 (100)	-
Total	396	391 (98.73)	5 (1.27)

**Table 2:** Number and size range of microplastic particles detected in long-tailed macaque fecal samples from Kosumpee Forest Park.

Macaque Group	Number of MPs (Pieces)	Size Range of MPs (%)					
		0–1 mm	1–2 mm	2–3 mm	3–4 mm	4–5 mm	>5 mm
DroopLip	81	52	18	7	2	2	-
RedDot	44	20	15	4	4	1	-
StumpTail	124	61	39	16	4	3	1
HareLip	31	12	13	6	-	-	-
BigRed	116	33	29	20	14	13	7
Total	396	178	114	53	24	19	8
%	100	44.95	28.78	13.38	6.06	4.79	2.02

## Data Availability

The data supporting this study are available within this article and could be further requested from the corresponding author.
